# RANK signaling increases after anti*-*HER2 therapy contributing to the emergence of resistance in HER2-positive breast cancer

**DOI:** 10.1186/s13058-021-01390-2

**Published:** 2021-03-30

**Authors:** Adrián Sanz-Moreno, Sonia Palomeras, Kim Pedersen, Beatriz Morancho, Tomas Pascual, Patricia Galván, Sandra Benítez, Jorge Gomez-Miragaya, Marina Ciscar, Maria Jimenez, Sonia Pernas, Anna Petit, María Teresa Soler-Monsó, Gemma Viñas, Mansour Alsaleem, Emad A. Rakha, Andrew R. Green, Patricia G. Santamaria, Celine Mulder, Simone Lemeer, Joaquin Arribas, Aleix Prat, Teresa Puig, Eva Gonzalez-Suarez

**Affiliations:** 1 Oncobell, Bellvitge Biomedical Research Institute (IDIBELL), L’Hospitalet de Llobregat, Barcelona, Spain; 2 Present Address: German Mouse Clinic, Institute of Experimental Genetics, HMGU, Neuherberg, 85764 Germany; 3 New Therapeutics Targets Lab (TargetsLab), Department of Medical Sciences, University of Girona, Girona, Spain; 4 Preclinical Research Program, Vall d’Hebron Institute of Oncology (VHIO), Barcelona, Spain; 5 Centro de Investigación Biomédica en Red de Cáncer (CIBERONC), Madrid, Spain; 6 Translational Genomics and Targeted Therapeutics in Solid Tumors, August Pi i Sunyer Biomedical Research Institute (IDIBAPS), Barcelona, Spain; 7 SOLTI Breast Cancer Research Group, Barcelona, Spain; 8 Department of Medical Oncology, Hospital Clinic, Barcelona, Spain; 9 Present Address: Department of Biomedicine, Department of Surgery, University Hospital Basel, University of Basel, Basel, Switzerland; 10 Molecular Oncology, Spanish National Cancer Research Centre (CNIO), Madrid, Spain; 11 Department of Medical Oncology, Breast Unit, Catalan Institute of Oncology (ICO), University Hospital of Bellvitge IDIBELL, L’Hospitalet de Llobregat, Barcelona, Spain; 12 Pathology Department, University Hospital of Bellvitge, IDIBELL, Barcelona, Spain; 13 Medical Oncology Department, Catalan Institute of Oncology (ICO), Girona, Spain; 14 Division of Cancer and Stem Cells, School of Medicine, University of Nottingham Biodiscovery Institute, University Park, Nottingham, NG7 2RD UK; 15 Biomolecular Mass Spectrometry and Proteomics Bijvoet Center, Utrecht University, Utrecht, The Netherlands; 16 Medicine Department, University of Barcelona, Barcelona, Spain

**Keywords:** Breast cancer, HER2, Lapatinib, NF-κB, RANK, RANKL, Resistance, Trastuzumab

## Abstract

**Background:**

Around 15–20% of primary breast cancers are characterized by HER2 protein overexpression and/or *HER2* gene amplification. Despite the successful development of anti-HER2 drugs, intrinsic and acquired resistance represents a major hurdle. This study was performed to analyze the RANK pathway contribution in HER2-positive breast cancer and anti-HER2 therapy resistance.

**Methods:**

RANK and RANKL protein expression was assessed in samples from HER2-positive breast cancer patients resistant to anti-HER2 therapy and treatment-naive patients. RANK and RANKL gene expression was analyzed in paired samples from patients treated with neoadjuvant dual HER2-blockade (lapatinib and trastuzumab) from the SOLTI-1114 PAMELA trial. Additionally, HER2-positive breast cancer cell lines were used to modulate RANK expression and analyze in vitro the contribution of RANK signaling to anti-HER2 resistance and downstream signaling.

**Results:**

RANK and RANKL proteins are more frequently detected in HER2-positive tumors that have acquired resistance to anti-HER2 therapies than in treatment-naive ones. *RANK* (but not *RANKL*) gene expression increased after dual anti-HER2 neoadjuvant therapy in the cohort from the SOLTI-1114 PAMELA trial. Results in HER2-positive breast cancer cell lines recapitulate the clinical observations, with increased RANK expression observed after short-term treatment with the HER2 inhibitor lapatinib or dual anti-HER2 therapy and in lapatinib-resistant cells. After RANKL stimulation, lapatinib-resistant cells show increased NF-κB activation compared to their sensitive counterparts, confirming the enhanced functionality of the RANK pathway in anti-HER2-resistant breast cancer. Overactivation of the RANK signaling pathway enhances ERK and NF-κB signaling and increases lapatinib resistance in different HER2-positive breast cancer cell lines, whereas RANK loss sensitizes lapatinib-resistant cells to the drug. Our results indicate that ErbB signaling is required for RANK/RANKL-driven activation of ERK in several HER2-positive cell lines. In contrast, lapatinib is not able to counteract the NF-κB activation elicited after RANKL treatment in RANK-overexpressing cells. Finally, we show that RANK binds to HER2 in breast cancer cells and that enhanced RANK pathway activation alters HER2 phosphorylation status.

**Conclusions:**

Our data support a physical and functional link between RANK and HER2 signaling in breast cancer and demonstrate that increased RANK signaling may contribute to the development of lapatinib resistance through NF-κB activation. Whether HER2-positive breast cancer patients with tumoral RANK expression might benefit from dual HER2 and RANK inhibition therapy remains to be elucidated.

**Supplementary Information:**

The online version contains supplementary material available at 10.1186/s13058-021-01390-2.

## Background

The human epidermal growth factor receptor 2 (HER2), known as ErbB2 or Neu, is a tyrosine kinase receptor protein encoded by the *ERBB2* (*HER2*) gene [1]. HER2 is a member of the epidermal growth factor (EGF) receptor family along with EGFR/HER1, ERBB3/HER3, and ERBB4/HER4. The four receptors are transmembrane proteins with an intracellular tyrosine kinase domain (although ERBB3/HER3 is considered kinase impaired). While HER2 is the only family member that does not bind to a ligand, it forms heterodimers with the other EGF receptor protein members and shows strong catalytic kinase activity, efficiently triggering downstream signaling through phosphatidylinositol-3 kinase (PI3K) and mitogen-activated protein kinase (MAPK) [1]. Approximately 15–20% of primary breast cancers show HER2 protein overexpression and/or *HER2* gene amplification [2], which is associated with poor prognosis. The development of humanized monoclonal antibodies binding the extracellular domain of HER2 (e.g., trastuzumab, pertuzumab), EGFR-HER2 small molecule kinase inhibitors (e.g., lapatinib, neratinib, or tucatinib), and antibody-drug conjugates (e.g., T-DM1 or DS-8201) has revolutionized HER2-positive breast cancer treatment [3]. Still, most patients with metastatic disease eventually progress on anti-HER2 therapy due to de novo or acquired resistance, and 20–30% of patients with early HER2+ breast cancer relapse [4–6]. Therefore, elucidating the mechanisms of resistance to anti-HER2 drugs is pivotal to further improve patients’ survival outcomes.

Receptor activator of nuclear factor kappa-Β ligand (RANKL) and its receptor (RANK) belong to the TNF superfamily. The fundamental role of RANK signaling in osteoporosis and bone metastasis inspired the development of denosumab, a monoclonal antibody against RANKL, for the treatment of skeletal-related events (SREs) linked to osteoporosis and cancer [7]. RANK signaling activation in the breast epithelium promotes tumor initiation, progression, and metastatic spread. Thus, RANK and RANKL have emerged as promising targets for breast cancer prevention and treatment [8]. RANKL is expressed in progesterone receptor-positive cells and acts as a paracrine mediator of progesterone in the mammary epithelia [9, 10]. Increased RANK receptor expression is more frequent in hormone receptor-negative tumors and high-grade breast cancer, but it is also found in a subset of luminal tumors [11, 12]. RANK signaling controls proliferation and stemness in BRCA1-mutant and oncogene-driven mammary tumors [13, 14]. Interestingly, RANK signaling inhibition has been shown to reduce HER2 tumorigenesis in preclinical studies [9, 15]. In human tumors, RANKL and HER2 levels predict metastasis to the bone in breast cancer better than RANKL alone [16].

Some of the common (intrinsic or acquired) resistance mechanisms to trastuzumab and/or lapatinib treatment are impaired HER2 binding, parallel/downstream pathway activation, ER signaling, cell cycle de-regulation, or escape from antibody-dependent cellular cytotoxicity (ADCC) [17]. Personalized treatment of HER2-positive breast cancer and better predictive biomarkers to anticipate therapy resistance will contribute to the identification of patients that will benefit from new combinatorial therapies, paving the way for HER2-positive breast cancer precision medicine [18].

In this study, we unveiled a functional relationship between RANK and HER2 signaling using HER2-positive breast cancer patient samples and cell lines. Upon analyses of HER2-positive breast cancer samples from treatment-naive patients and residual disease at surgery after neoadjuvant anti-HER2 therapy, including paired samples from the phase II SOLTI-1114 PAMELA trial, we observed that anti-HER2 treatment or resistance to anti-HER2 therapy both resulted in increased RANK expression. Additionally, when we analyzed the effects of RANK modulation on anti-HER2 treatment in HER2-positive breast cancer cell lines, we observed that enhanced RANK signaling led to increased lapatinib resistance.

## Methods

### Patient samples

RANK and RANKL expression was assessed in tumor samples from three different cohorts of patients with HER2-positive breast cancer.

#### Treatment-naive cohort

Patients with primary and operable HER2-positive breast cancer (*n* = 197) diagnosed from 2003 to 2010 at the Nottingham City Hospital, Nottingham, UK. Tumor samples were collected at surgery prior to any neoadjuvant treatment. Histological grade was assessed by the Nottingham Grading System [19] and other clinicopathological factors such as tumor size, lymph node (LN) status, estrogen receptor (ER), progesterone receptor (PR), and HER2 expression, as well as patient age and disease progression, were analyzed before including the samples into the TMAs, prepared as previously described [20].

#### Anti-HER2-resistant cohort

Patients treated with trastuzumab-based primary chemotherapy and residual disease at surgery (*n* = 43) diagnosed at the Catalan Institute of Oncology (ICO), Bellvitge University Hospital in l’Hospitalet de Llobregat, and Dr. Josep Trueta University Hospital in Girona (Spain) between 2005 and 2014 and described in [21]. The selection criterion included patients with early or locally advanced HER2-positive breast cancer (including inflammatory breast cancer) who had received neoadjuvant treatment with trastuzumab-based chemotherapy and had residual invasive disease following surgery (i.e., who had not achieved a pathological complete response at surgery). Neoadjuvant chemotherapy was based on anthracyclines and taxanes given concurrently with weekly trastuzumab for 24 weeks followed by surgery. For all patients, hematoxylin and eosin (H&E)-stained slides from formalin-fixed paraffin-embedded (FFPE) tumor blocks were examined to determine representative areas of the invasive tumor. ER, PR, and HER2 positivity were assessed in the initial tumor core biopsies as well as in the residual disease. For each patient, different clinical and histopathological features such as age, and histological grade (Nottingham Grading System) were obtained.

#### SOLTI-1114 PAMELA cohort

Patients treated with neoadjuvant dual-blockade trastuzumab and lapatinib (*n* = 151) and in which biopsy paired samples were prospectively obtained. The main results of the PAMELA neoadjuvant phase II study have been previously reported [22] and the completed study is registered in ClinicalTrials.gov (number NCT01973660). In this trial, patients with early HER2-positive breast cancer were treated with neoadjuvant lapatinib (1000 mg daily) and trastuzumab (8 mg/kg i.v. loading dose followed by 6 mg/kg) for 18 weeks. Patients with hormonal receptor-positive breast cancer received letrozole or tamoxifen according to menopausal status. FFPE tumor samples at baseline, at day 14 of treatment, and at surgery were collected according to standard protocols.

### Gene expression analyses

RNA samples of the PAMELA trial from tumors at baseline (*n* = 151) were previously analyzed [22]. Here, the nCounter platform (NanoString Technologies, Seattle, WA, USA) analyzed RNA of 101 residual tumors from surgical samples of the PAMELA trial. A minimum of 100 ng of total RNA was used to measure the expression of 550 genes, including *RANK* and *RANKL*, and 5 housekeeping genes (*ACTB*, *MRPL19*, *PSMC4*, *RPLP0*, and *SF3A1*). Expression counts were then normalized using the the nSolver 4.0 software and custom scripts in R 3.4.3. For each sample, we calculated the PAM50 signature scores (basal-like, HER2-E, luminal A and B, normal-like) and the risk of recurrence score [23]. Intrinsic molecular subtypes were identified using the research-based PAM50 predictor as previously described [22, 24].

### Immunohistochemistry and tissue microarray scoring

Immunohistochemistry (IHC) in TMAs was performed using anti-human mouse monoclonal RANK (N-1H8 Amgen) and human RANKL (M366 Amgen) as described in [9]. RANK or RANKL staining was scored on a scale of 0 to 3 for intensity (0 = no staining, 1 = weak, 2 = moderate, 3 = intense) and for the percentage of positively stained tumor cells (0–100) as previously reported [25]. The result of multiplying staining intensity by positive cell percentage is the *H*-score value, ranging from 0 to 300. TMA cores were scored for RANK and RANKL with the assistance of the breast cancer pathologists from the Bellvitge Hospital, if tumor cells represented > 15% of the total TMA core area. Patients were stratified according to RANK or RANKL *H*-scores as being protein-positive (*H*-score ≥ 1) or protein-negative (*H*-score = 0). Breast tumors from patient-derived xenografts were used as positive and negative controls. Experimental data from our laboratory in breast cancer cells and patients’ samples [26] confirmed that cells in which RANK protein expression is not detected by IHC/western blot may still respond to RANKL stimulation or denosumab inhibition [11, 26, 27]. This is probably due to the “fragility” of the RANK epitope and the limited sensitivity of the current tools to detect RANK protein expression. Thus, even with an *H*-score ≥ 1, we are likely underestimating samples with a functional RANK signaling pathway.

Statistical analyses were performed with the support of IDIBELL and Nottingham University Statistical Assessment Services. The ER/PR/HER2 status, grade, and tumor stage were known for each case included in the TMAs. Associations between IHC scores and clinicopathological parameters were evaluated using Pearson’s chi-squared test.

### Cell lines and cell culture

The cell lines BT474 parental (BT474) and BT474 with lapatinib resistance (BTLR) were described in [28]. SKBR3 parental (SKBR3) and SKBR3 lapatinib resistant (SKLR) lines were described in [29]. The cell line HCC1954 was obtained from ATCC (CRL-2338). BT474 cells were grown in DMEM + GlutaMAX (Gibco) supplemented with 2 mM l-glutamine (HyClone), 1× penicillin-streptomycin solution (P/S, Gibco), and 7.5% fetal bovine serum (FBS, Gibco). SKBR3 cells were grown in McCoy’s 5A + GlutaMAX supplemented with 2 mM l-glutamine, 1 mM sodium pyruvate (HyClone), 1× P/S, and 5% FBS. HCC1954 cells were grown in RPMI medium 1640 + GlutaMAX supplemented with 2 mM l-glutamine, 1× P/S, and 5% FBS. The cells were grown at 37 °C in 5% CO_2_ humidified incubators. For RANKL treatments, cells were incubated in the presence of 100–300 ng/ml of RANKL. Cell lines were routinely tested for mycoplasma contamination.

### Viral transduction

To ectopically express RANK, the *RANK* gene (*TNFRSF11A*) was cloned into the lentiviral vector pSD-69 (kindly provided by S. Duss and M. Bentires-Alj) under the control of hPGK promoter. As a control (ctrl), we used an empty pSD-69 plasmid generated by removing the BamHI-SalI fragment containing CcdB and CmR genes. Knockdown of *RANK* endogenous expression was achieved by shRNA lentiviral delivery using pGIPZ vectors containing shRNAs against human *RANK* (RHS4531, Dharmacon), and shRNAs sequences #3 (TATCTTCTTCATTCCAGCT) and #4 (ATTCTTCCTTGAACTTCCC) were selected based on their ability to silence *RANK* expression. As a control, we used pGIPZ expressing a verified non-targeting sequence (RHS4346 Dharmacon). Lentiviruses were prepared in HEK293T cells transfected with psPAX2 (Addgene #12260) and pMD2.G (Addgene #12259) by the calcium phosphate method. Virus-containing supernatants were centrifuged at 1500 rpm for 5 min and filtered with 0.45-μm filters (Millipore). The medium from 1-cm^2^ production cells was used to infect 2-cm^2^ recipient cells at roughly 33% confluence, adding fresh medium (1:1) and 8 μg/ml polybrene (Millipore). Approximately 90% infection efficiency was verified 3 days after transduction by detection of GFP expressed from pGIPZ plasmids. Transduced cells were selected with 1.5 μg/ml puromycin (Sigma), starting 3 days after infection, and subsequently maintained with 1 μg/ml puromycin in the growth media.

### Cell proliferation

To determine cell proliferation, 1000–4000 cells per well in 100 μl were seeded in 96-well plates. After 24 h, 100 μl of medium with or without the indicated concentrations of lapatinib (0–16 μM) was added, and cells were incubated for 4 days. The relative number of viable cells each day was determined by adding 50 μl of diluted CCK-8 reagent according to the manufacturer’s protocol (Sigma).

### Western blot

Cells were seeded at approximately 33% confluence in 6-well plates. The following day, they were washed and incubated in a medium without FBS. The next day, the medium was changed to 1.8 ml medium with or without 1 μM lapatinib followed by a 2-h incubation. Subsequently, 0.2 ml of medium with or without 300 ng/ml of RANKL (RANKL-LZ Amgen) was added to the wells. Ten minutes later, the extracts for immunoblots were prepared with modified RIPA buffer (50 mM Tris pH 7.4, 150 mM NaCl, 1% NP-40, 0.25% sodium deoxycholate) containing 1× PhosSTOP and complete protease inhibitor cocktail (Roche), and protein concentrations determined with DC protein assay reagents (BIO-RAD). Fifteen micrograms of protein were resolved by SDS-PAGE and blotted into Immobilon-P 0.45 μm membranes (Millipore). Antibodies against the following proteins were used for probing: RANK (R&D Systems AF683), p-HER2 (#2249), HER2 (#2165), p-EGFR (#3777), EGFR (#4267), p-ERK1/2 (#9101), ERK1/2 (#9102), p-AKT (#4051), AKT (#9272), p-p65 (#3033), p65 (#8242), p-IκB (#9246), IκB (#9242) (from Cell Signaling), β-actin (sc-47778), and tubulin (Abcam ab21058).

### Immunoprecipitation

Upon transiently transfecting HEK293 cells with affinity-tagged versions of full-length RANK (RANK-V5 in pLenti6/V5-DEST, Invitrogen), full-length HER2 (FLAG-HER2 [30]), an amino (742-NTF) [30], or carboxy-terminal fragment of HER2 (611-CTF) [31], cells were washed twice with ice-cold PBS and proteins were extracted with 20 mM Tris-HCl pH 7.4, 137 mM NaCl, 2 mM EDTA, 10% glycerol, 1% NP-40 supplemented with 50 μg/ml leupeptin, 50 μg/ml aprotinin, 0.5 mM sodium orthovanadate, and 1 mM phenylmethylsulfonyl fluoride. Equal amounts of extracts were incubated for 3 h with immunoglobulin G (Abcam ab171870), FLAG (Sigma F3165), HA (Abcam ab9110), V5 (Thermo Scientific #R961-25), HER2 (32H2 in house antibody described in [32]), or trastuzumab (Hoffmann-La Roche) antibodies. Then, protein A agarose beads (Calbiochem IP02) were added for 2 h. Immunoprecipitates were washed thoroughly with lysis buffer and boiled in reducing SDS loading buffer to be analyzed by Western blot.

### RNA isolation and RT-qPCR

Cells were seeded at approximately 33% confluence in 6-well plates. The next day, the medium was changed to medium with or without 100 ng/ml RANKL followed by an additional 24 h incubation period. To analyze mRNA expression levels, total RNA was purified with Maxwell RSC simplyRNA Tissue kit (AS1340 Promega). For each sample, cDNA was retrotranscribed from 1 μg of RNA using 200 U SuperScript II plus random hexamer oligos following the manufacturer’s protocol (Invitrogen); cDNA from 20 ng RNA for each sample was analyzed by SYBR green real-time PCR (Applied Biosystems) with 10 μM primers using a LightCycler® 480 thermocycler (Roche). Analyses were performed in triplicates using the LightCycler® 480 software (Roche). Peptidylprolyl isomerase A, *PP1A*, was used as the reference gene. The primer sequences used in the analyses are as follows: *PP1A* (fw ATGGTCAACCCCACCGTT, rev TCTGCTGTCTTTGGGACCTTG), *RANK* (fw GCAGGTGGCTTTGCAGAT, rev 5’GCATTTAGAAACATGTACTTTCCTG), *BIRC3* (fw GGTAACAGTGATGATGTCAAATG, rev TAACTGGCTTGAACTTGACG), *ICAM1* (fw AACTGACACCTTTGTTAGCCACCTC, rev CCCAGTGAAATGCAAACAGGAC), *TNFα* (fw AAGCTGTAGCCCATGTTGT, rev TGAGGTAACAGGCCCTCTGAT), and *IL8* (fw CTGCGCCAACACAGAAATTA, rev CATCTGGCAACCCTACAACA).

## Results

### RANK is expressed in HER2-positive and anti-HER2-resistant breast cancer patients

The expression of RANK and RANKL in HER2-positive breast cancer patients was analyzed by immunohistochemistry (IHC) in two independent sets of tissue microarrays (TMAs): a collection of HER2-positive tumor samples from treatment-naive patients (*n* = 197) and a cohort with tumors resistant to neoadjuvant trastuzumab-based chemotherapy (*n* = 43) from patients with residual invasive disease at surgery.

**Fig. 1:**
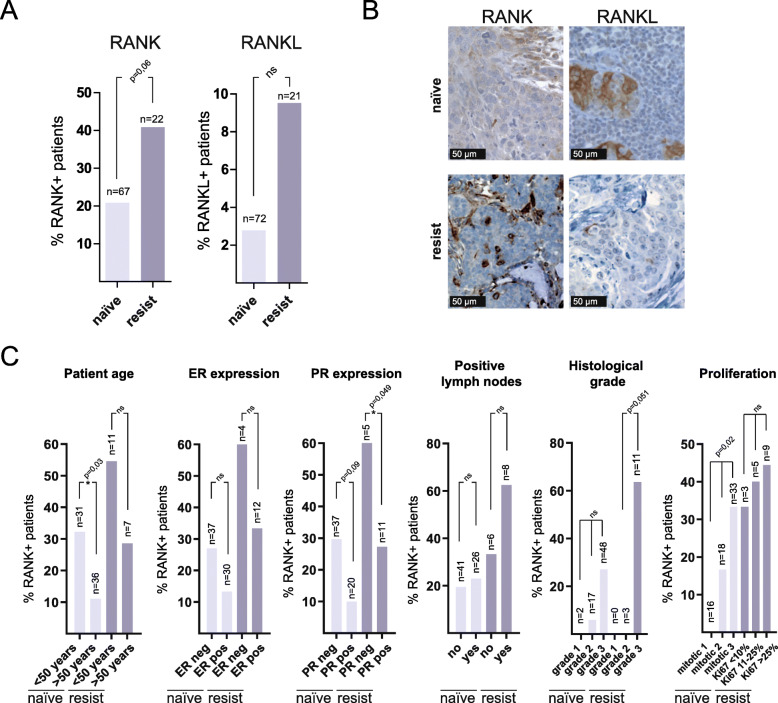
RANK and RANKL are expressed in treatment-naive and anti-HER2-resistant HER2 breast cancer tumor samples. **a** Frequency of HER2-positive patients, treatment-naive or anti-HER2-resistant, expressing tumoral RANK or RANKL (H-score ≥ 1). The total number of patients scored for RANK or RANKL expression is indicated. **b** Representative images showing RANK and RANKL IHC. **c** Frequency of tumoral RANK-positive treatment-naive HER2 or anti-HER2-resistant patients and associations with the indicated clinicopathological parameters including those assessed by the Nottingham Grading System (histological grade and proliferation determined by mitotic count and Ki67 as detailed in [19]). The total number of patients analyzed per parameter is indicated in each case. In **a** and **c**, two-sided chi-square *p* values are shown when *p* < 0.1. n.s., non-significant (see also Fig. S1A)

In the first collection, the integrity of the tissue allowed scoring of 67 and 72 patients for RANK and RANKL expression, respectively. Considering positive those with *H*-score ≥ 1 for the tumor cells, RANK expression was found in 14/67 (20.9%) cases and transmembrane RANKL staining in just 2/72 (2.8%) of the samples (Fig. 1a). In the anti-HER2-resistant tumor samples, we could score 22 patients for RANK and 21 for RANKL (Fig. 1a). In these, 9/22 (40.9%) were positive for RANK and 2/21 (9.5%) for transmembrane RANKL in tumor cells (Fig. 1a). Representative pictures of RANK and RANKL positive samples are shown in Fig. 1b, and *H*-scores for the whole tumor core from all samples (excluding those with integrity issues) and controls are presented in Fig. S1A and B. Pictures of the whole TMA core area in both collections are shown in Fig. S2.

Next, we evaluated the clinico-pathological factors associated with RANK expression in treatment-naive HER2-positive tumors (Fig. 1c). RANK expression was significantly associated with tumors from younger patients (less than 50 years old; *p* = 0.034) and tumors with a higher Ki67 proliferation index (*p* = 0.02). A trend of increased frequency of RANK expression was found in ER/PR negative tumors (*p* = 0.170 and *p* = 0.090, respectively), and higher histological grade (*p* = 0.138) (Fig. 1c). Similar patterns were observed in tumors resistant to anti-HER2 treatment (Fig. 1c). In both series, the limited number of samples prevented additional statistically significant associations, but general patterns coincided with those reported in previous studies of RANK/RANKL expression in human breast cancer samples [11, 12, 33]. Importantly, the frequency of RANK/RANKL-positive samples was higher in anti-HER2-resistant compared to treatment-naive HER2-positive tumors.

### RANK expression increases after anti-HER2 treatment in HER2-positive breast cancer patients (PAMELA clinical trial)

**Fig. 2:**
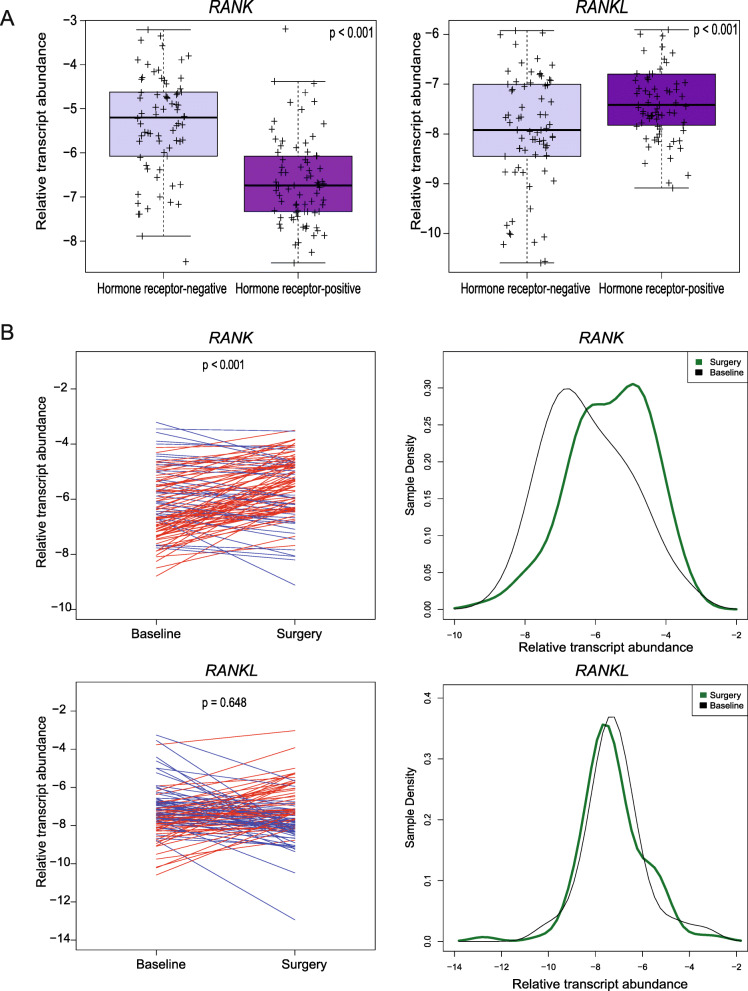
*RANK,* but not *RANKL,* expression increased after dual anti-HER2 therapy in patient samples (*n* = 151) from the PAMELA trial. **a** Box plots of *RANK* and *RANKL* gene expression in HER2-positive tumors at baseline classified by hormone receptor expression. **b** Ladder plots (on the left) show the *RANK* and *RANKL* gene expression in PAMELA HER2-positive tumors before (baseline) and after (surgery) dual anti-HER2 treatment. An increase in gene expression is represented in red and a decrease in blue. Each line represents a tumor sample from one patient. *p* values in **a** were calculated by comparing the mean values between both groups and in **b** were determined by paired two-tailed *t* tests. Density plots (on the right) showing the *RANK* and *RANKL* gene expression in PAMELA HER2-positive tumors before (baseline) and after (surgery) treatment (see Fig. S3, S4, and Supplementary Table 1 for further analyses)

Our previous results suggested that RANK and RANKL expression may increase upon acquisition of anti-HER2 treatment resistance (Fig. 1a). To determine the possible changes in RANK and RANKL expression induced by dual HER2 blockade, gene expression profiling was performed in paired surgical tumor samples obtained before and following treatment with lapatinib and trastuzumab (and endocrine therapy if the tumor was hormone receptor-positive) from the PAMELA phase II clinical trial [22]. At baseline, the expression of *RANK* was significantly associated with the PAM50 intrinsic subtypes (Fig. S3A; *p* < 0.001); non-luminal subtypes (Basal-like and HER2-enriched) had the highest *RANK* expression. No significant differences in *RANKL* gene expression across PAM50 intrinsic subtypes were observed, although *RANKL* levels were slightly increased in the luminal A subtype (Fig. S3A), as previously reported [33]. Moreover, *RANK* gene expression was higher in hormone receptor-negative tumor samples (*p* < 0.001) while *RANKL* showed the opposite trend (Fig. 2a) confirming previous findings [12, 34]. *ERBB2* gene expression at baseline had a weak positive correlation (*r* = 0.16) with *RANK* and the opposite trend (*r* = − 0.21) with *RANKL* expression (Fig. S3B and C). *RANK* gene expression increased (Fig. 2b, denoted by red lines in the left graph and a green line in the right graph) following dual treatment with lapatinib and trastuzumab (*p* < 0.001), while *RANKL* expression did not significantly change when analyzing residual disease samples at surgery (Supplementary Table 1). Specifically, the mean *RANK* expression in baseline samples was − 6.22 (standard deviation (SD) = 1.22) versus − 5.58 (SD = 1.14) in the paired surgical samples. In contrast, the mean *RANKL* expression in the baseline samples was − 7.36 (SD = 1.28) versus − 7.44 (SD = 1.33) in the surgical samples. When populations were studied separately according to hormone receptor expression, the same findings were observed, an increase in *RANK* mRNA expression in both hormone receptor positive and negative HER2+ tumors after HER2 inhibition (Fig. S4A and B).

These results confirmed that *RANK* expression increases in HER2-positive breast tumors after dual HER2 blockade. The increased levels of RANK observed in patients upon anti-HER2 treatment suggest that activation of RANK signaling may allow survival of HER2-positive tumor cells and contribute to resistance to anti-HER2 therapies.

### RANK signaling is upregulated after short-term lapatinib treatment and in HER2-resistant cell lines

As RANK expression increased after dual lapatinib/trastuzumab treatment in HER2-positive breast cancer patients, we decided to test whether in vitro short-term treatment with both anti-HER2 drugs, alone or in combination, would influence RANK expression in three different HER2-positive breast cancer cell lines. While SKBR3 and BT474 cells are sensitive to lapatinib and trastuzumab, HCC1954 cells are less sensitive to lapatinib and resistant to trastuzumab [35].

**Fig. 3:**
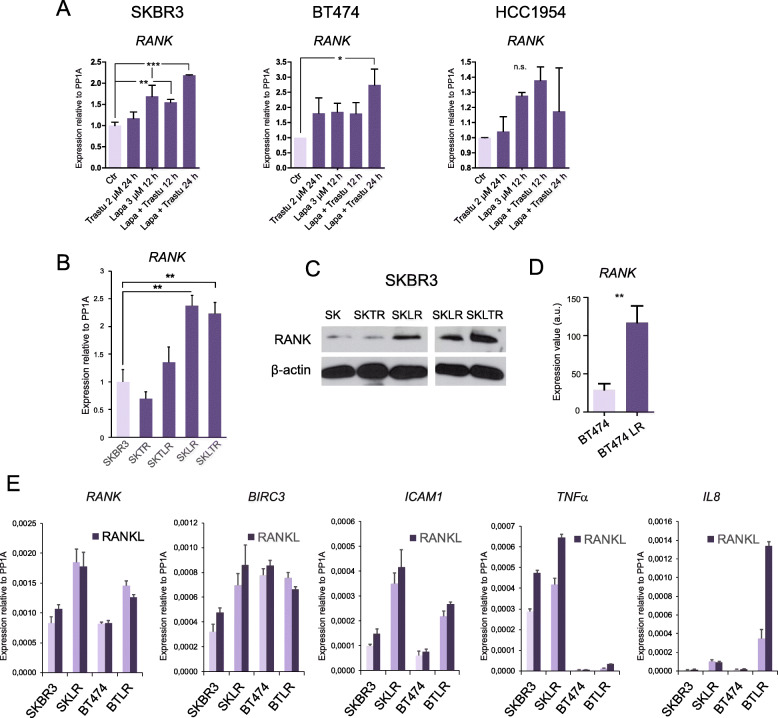
RANK expression increased in HER2-positive breast cancer cell lines after treatment with anti-HER2 therapies as well as in anti-HER2-resistant cells. **a** *RANK* gene expression levels determined by RT-qPCR in the indicated HER2-positive cell lines after short-term treatment with lapatinib (Lapa), trastuzumab (Trastu), or the combination of both, relative to corresponding untreated cells (Ctr). Quantifications were performed in triplicates from two independent experiments. **b** *RANK* gene expression levels determined by RT-qPCR in SKBR3 cell lines resistant to trastuzumab (SKTR), lapatinib (SKLR), or both drugs (SKTLR, SKLTR) compared to sensitive SKBR3 parental cells. Quantifications were performed in triplicates from at least three independent experiments. **c** RANK protein expression in parental SKBR3 cells (SK) or resistant to trastuzumab (SKTR), lapatinib (SKLR), or both (SKLTR). β-Actin was used as a loading control. Blots shown are representative of those obtained from 3 independent experiments. **d** *RANK* gene expression levels in BT474 cells, either control or resistant to lapatinib (LR, according to public datasets [36]). a.u., arbitrary units. **e** *RANK* and RANK/NF-κB downstream gene targets *BIRC3*, *ICAM1*, *TNFα*, and *IL8* mRNA levels relative to housekeeping gene *PP1A* in parental (SKBR3, BT474) and lapatinib-resistant (SKLR, BTLR) HER2-positive cell lines with or without RANKL treatment (24 h). Gene expression levels were quantified by RT-qPCR. *PP1A* expression was used as an internal reference gene (**a**, **b**, **e**). Determinations were done in triplicates, and the mean values are depicted from *n* ≥ 2 independent analyses. *p* values were calculated by ordinary one-way ANOVA (**a**, **b**) and by unpaired *t* tests (**d**) (*≤ 0.05; **≤ 0.01, ***≤ 0.001; n.s., non-significant)

Lapatinib treatment, alone or in combination with trastuzumab, resulted in higher *RANK* mRNA expression in SKBR3 when compared with non-treated cells (Fig. 3a). Lapatinib or trastuzumab treatment, as well as their combination, also increased *RANK* expression levels in BT474 cells. In HCC1954 cells, *RANK* expression increased with lapatinib alone or in combination treatment after 12 h, whereas trastuzumab alone did not alter *RANK* expression levels. Also, we analyzed *RANK* expression in SKBR3 cells, either parental (sensitive to lapatinib and trastuzumab) or resistant to trastuzumab (SKTR), to lapatinib (SKLR), or to both (SKTLR and SKLTR; derived from SKTR and SKLR, respectively) [29]. RANK gene and protein expression levels were significantly higher in lapatinib-resistant SKLR and dual lapatinib/trastuzumab-resistant SKLTR cells when compared to SKBR3 parental cells (Fig. 3b, c). Increased *RANK* mRNA expression was also observed in BT474 cells with acquired lapatinib resistance (LR) when compared to lapatinib-sensitive parental cells according to public datasets, platform ID: GPL570 [36]; (Fig. 3d), an increase we verified by RT-qPCR (Fig. 3e).

To confirm that the elevated *RANK* expression levels were accompanied by increased activation of the RANK signaling pathway, the expression of RANK downstream gene targets *BIRC3*, *ICAM1*, *TNFα*, and *IL8*, indicative of NF-κB pathway activation [37, 38], was analyzed in sensitive and lapatinib-resistant (LR) cells treated with or without RANKL. Lapatinib-resistant SKLR cells showed higher gene expression levels of *RANK*, *BIRC3*, *ICAM1*, *TNFα*, and *IL8* compared with control SKBR3 cells, and their levels were further increased after pathway stimulation with RANKL, except for *IL8* (Fig. 3e). In lapatinib-resistant BTLR, increased expression of *RANK* and its downstream targets *ICAM1* and *IL8* was detected, and their levels increased further upon RANKL stimulation compared to sensitive BT474 cells. In these cells, *BIRC3* expression did not change whereas *TNFα* was barely expressed (Fig. 3e).

Taken together, *RANK* expression increased after dual treatment with lapatinib and trastuzumab in HER2-positive human breast cancer cell lines, mimicking the results seen in breast cancer samples from the PAMELA trial (Fig. 2b). Additionally, two HER2-positive cell lines (SKBR3 and BT474) with acquired resistance to lapatinib (SKLR and BTLR) showed increased expression of RANK and several downstream targets, when compared to their respective parental controls (Fig. 3b–e).

### RANK overactivation increases NF-κB signaling and resistance to lapatinib

**Fig. 4:**
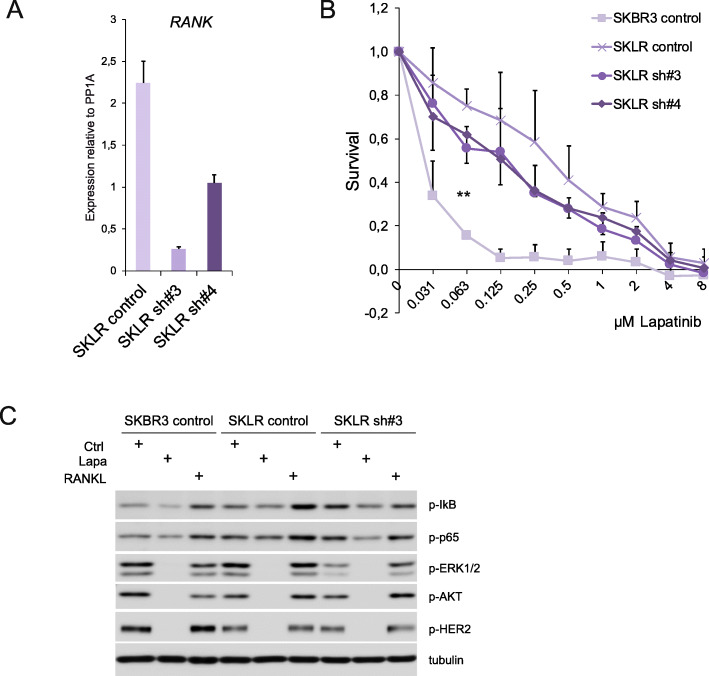
RANK knockdown slightly resensitizes SKLR cells to lapatinib. **a** The expression levels of *RANK* mRNA in lapatinib-resistant SKLR cells stably transduced with non-targeting (control) or two independent RANK knock-down (sh#3 and #4) vectors. *RANK* expression values were quantified by RT-qPCR relative to *PP1A* gene expression. Quantifications were performed in triplicates. **b** Relative number of living (relative survival) cells stably transduced with control (SKBR3 and SKLR), sh#3 or sh#4 (SKLR) and incubated for 4 days with the indicated concentrations of lapatinib. Cells were seeded in growth media; 24 h later lapatinib was added and cells were analyzed with CCK8 as detailed in the “Methods” section. The mean values and SD of four independent experiments are shown. For each experiment, data was obtained from quintuplicates. Paired *t* tests were done between the groups, and the two-tailed *p* value is depicted (**). In accordance with the lower expression of RANK achieved, sh#3 significantly reduced survival compared to SKLR control cells at 0.063 (*p* = 0.0097), 0.125 (*p* = 0.0055), and 0.25 (*p* = 0.0003) μM of lapatinib. For sh#4, a significant reduction in survival was observed at 0.125 μM of lapatinib (*p* = 0.014). The significance of relative survival was calculated for each concentration using two-tailed *p* values for one sample *t* test. **c** Western blot showing the levels of NF-κB (p-p65, p-IκB) and HER2 (p-HER2, p-ERK1/2, p-AKT) pathway activation in control SKBR3, lapatinib-resistant SKLR, and sh#3 SKLR cells treated with RANKL or lapatinib. Cells were serum-starved for 12 h and then treated with lapatinib (2 h) or RANKL (10 min) before processing them. Tubulin was used as a loading control (see Fig. S5B for total protein levels and Fig. S5C for relative quantifications)

To verify that RANK plays a direct role in the cellular response to lapatinib, we studied the consequences of RANKL stimulation and RANK loss in control and lapatinib-resistant SKBR3 cell lines. A small increase in lapatinib tolerance was observed in SKLR but not SKBR3 cells in the presence of RANKL (Fig. S5A). RANK silencing with two specific shRNAs reduced lapatinib resistance in SKLR cells, although sensitivity was not fully restored to the levels observed in WT cells (Fig. 4a, b). These results indicate that the activation of RANK signaling contributes to lapatinib resistance; however, it is not the only mechanism responsible for the emergence of resistance in SKLR cells. This is in line with the multiple anti-HER2 resistance mechanisms reported for these cells [39–41].

Increased IκB and p65 phosphorylation was observed in lapatinib-resistant SKLR compared to SKBR3 cells upon RANKL stimulation (Fig. 4c, Fig. S5B and C), confirming the elevated NF-κB signaling in lapatinib-resistant cells. As expected, RANKL-induced NF-κB activation was abrogated upon RANK silencing in SKLR cells. RANKL treatment did not significantly alter the phosphorylation status of AKT nor ERK in SKBR3, SKLR, and the RANK-silenced cells (Fig. 4c, S5B and C). After lapatinib treatment, HER2, AKT, and ERK1/2 protein phosphorylation levels were undetectable in all cell lines, but baseline phosphorylation of p65 and IκB was maintained (Fig. 4c, S5B and C), demonstrating that NF-κB activation is not dependent on ErbB signaling and may support the survival of HER2-positive breast cancer cells in the presence of lapatinib.

**Fig. 5:**
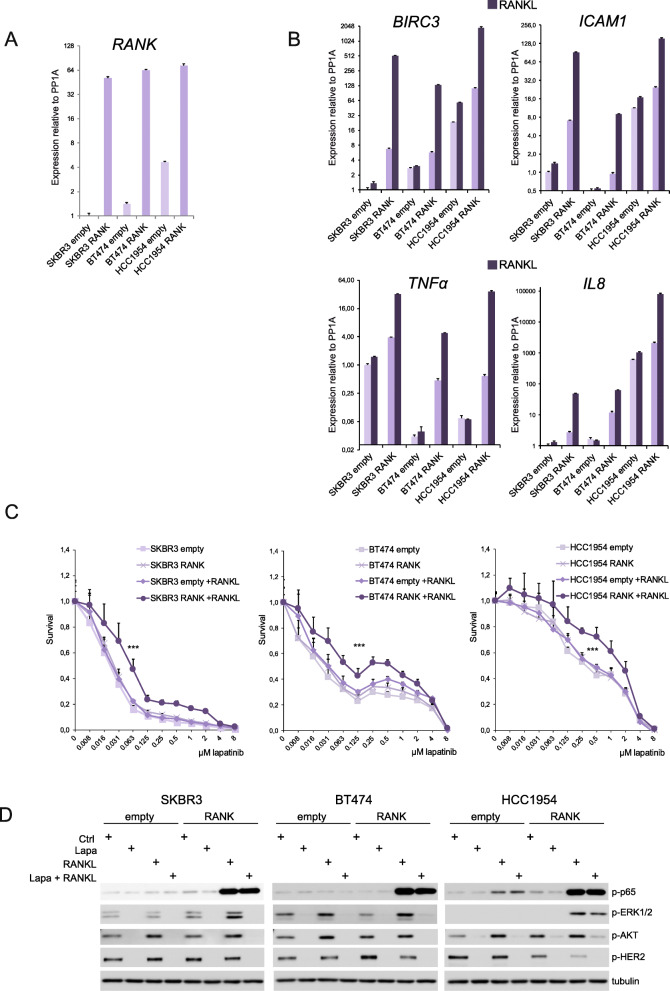
Overactivation of RANK signaling in HER2-positive cell lines increased NF-κB activation and lapatinib resistance. **a** Expression levels of *RANK* mRNA in HER2-positive SKBR3, BT474, and HCC1954 cells stably transduced with control (empty) or RANK-overexpressing (RANK) vectors. *RANK* expression values were quantified by RT-qPCR relative to *PP1A* gene expression. Experiments were performed in triplicates and standard error is depicted. **b** Expression levels of RANK/NF-κB downstream gene targets *BIRC3*, *ICAM1*, *TNFα*, and *IL8* relative to *PP1A* gene expression in cells described in **a**, with and without RANKL treatment (24 h). Experiments were performed in triplicates and standard error is depicted. **c** Relative number of living (relative survival) SKBR3, BT474, and HCC1954 cells stably transduced with control (empty) or RANK-overexpressing (RANK) vectors incubated for 4 days with the indicated concentrations of lapatinib and/or stimulated with RANKL. Cells were seeded in growth media with/without 100 ng/ml RANKL; 24 h later, lapatinib was added and cells were analyzed with CCK8 after 4 days as detailed in the “Methods” section. A representative experiment out of three independent experiments is shown. For each experiment, data was obtained from triplicates and SD, and a two-way ANOVA *p* value is included. **d**. Western blot analyses of NF-κB (p-p65) and HER2 (p-HER2, p-ERK1/2, and p-AKT) pathway activation in cells depicted in **c**. Before collecting the cells, they were cultured in media without FBS for 12 h and pretreated with/without lapatinib for 2 h followed by 10 min stimulation with RANKL. Representative blots from three independent experiments are shown. Tubulin was used as a loading control (see Fig. S6B for total protein levels, Fig. S6C for quantifications and Fig. S7 for EGF/HRG stimulations)

To test if RANK signaling and enhanced NF-κB activation may directly contribute to resistance, we stably transduced HER2-positive cell lines with RANK overexpressing (psD69-RANK) and empty control (psD69-empty) vectors. RANK overexpression was confirmed by increased *RANK* mRNA levels (Fig. 5a) and induction of NF-κB downstream targets (*BIRC3*, *ICAM1*, *TNFα*, and *IL8*) in SKBR3, BT474, and HCC1954 cells (Fig. 5b). These RANK-overexpressing cell lines showed enhanced expression of all NF-κB targets analyzed after RANKL treatment compared to the corresponding parental cells (Fig. 5b). Next, we tested whether increased activation of RANK signaling would alter the cell response to lapatinib; RANKL stimulation of control cells (empty) did not alter lapatinib sensitivity (Fig. 5c). In contrast, RANK overexpression coupled with RANKL treatment resulted in an increased resistance to lapatinib in all HER2-positive cell lines tested (Fig. 5c), and this effect was abrogated by the RANKL inhibitor denosumab as expected (Fig. S6A).

We then analyzed RANK downstream signaling in these cell lines after treatment with lapatinib and/or RANKL. p65 was strongly phosphorylated in RANK-overexpressing cell lines upon RANKL treatment and in parental HCC1954 cells fitting with the higher RANK expression levels of these cells (Fig. 5d, Fig. S6B and C). Phosphorylation of p65 was not affected by lapatinib treatment. ERK1/2 phosphorylation levels increased after RANKL treatment to a greater extent in the RANK-overexpressing cells compared to control ones (Fig. 5d, Fig. S6B and C). AKT phosphorylation increased after RANKL stimulation in all cells irrespectively of RANK levels. Interestingly, RANKL-mediated activation of ERK1/2 and AKT in SKBR3 and BT474 cells overexpressing RANK was completely abrogated in the presence of lapatinib, meaning that ErbB signaling is required for RANK/RANKL-driven activation of ERK and AKT in these cells. In HCC1954 cells, AKT phosphorylation was also abolished by lapatinib. In contrast, the increased p-ERK levels upon RANKL stimulation in HCC1954 RANK-overexpressing cells were not affected by lapatinib (Fig. 5d, Fig. S6B and C).

In summary, enhanced RANK signaling in HER2-positive cells led to higher NF-κB activation and increased lapatinib resistance.

### RANK and HER2 physically and functionally interact

To investigate whether RANK/RANKL activation of ERK and AKT might take place, at least partially, via direct crosstalk with ErbB receptors, we compared the phosphorylation levels of HER2 in cells with and without RANK overexpression upon RANKL stimulation. RANK overexpression led to higher levels of p-HER2 in SKBR3 and BT474, but not in HCC1954 cells, compared with the corresponding controls (Fig. 5d, Fig. S6B and C). Importantly, in all HER2-positive cell lines, concomitant RANK overexpression and stimulation with RANKL resulted in decreased HER2 phosphorylation, indicating that RANKL might impinge on the HER2/ErbB signaling pathway (Fig. 5d).

To further study the putative crosstalk between RANK and ErbB signaling, we analyzed NF-κB and ErbB signaling after stimulation with ErbB ligands in RANK-overexpressing HER2-positive cell lines and corresponding controls at different time points. A slight increase in p65 phosphorylation was observed in SKBR3 and BT474 RANK-overexpressing cells compared with control cells (Fig. S7). EGF stimulation faintly increased p-p65 levels in HER2-positive cell lines, but this was not observed after heregulin (HRG) treatment (Fig. S7). As extensively reported [42], treatment with EGF and HRG efficiently induces ERK phosphorylation in all HER2-positive cell lines (Fig. S7), but no clear differences were observed between RANK-overexpressing cells and corresponding controls. Of note, 5 min after ErbB ligand stimulation, pERK levels are higher but a decrease in HER2 phosphorylation was observed, accompanied by less pERK and pHER2 after 10 min of ErbB ligand stimulation (Fig. S7). Thus, the reduced HER2 phosphorylation observed in RANK-overexpressing cells 10 min after RANKL stimulation may reflect previous activation of HER2/ERK signaling.

**Fig. 6:**
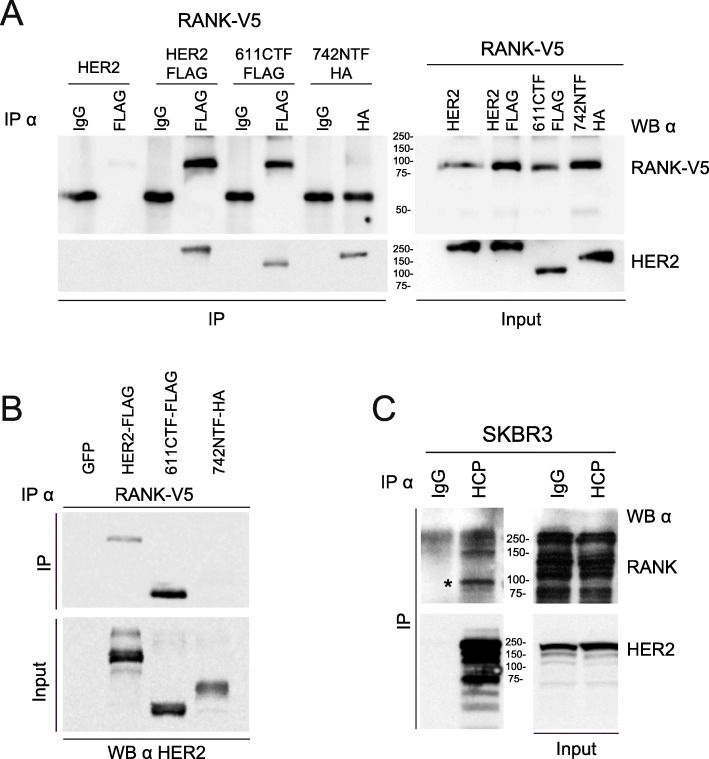
Co-immunoprecipitation of RANK and HER2. **a** Immunoprecipitation (IP) against HER2 was performed in HEK293 cells transfected with RANK-V5 and HER2, HER2-FLAG, a carboxy-terminal fragment of HER2 (611-CTF) or an amino-terminal fragment of HER2 (742-NTF). IP was performed using anti-FLAG, anti-HA, or control IgG antibodies as indicated. RANK was detected by blotting the immunoprecipitates (IP, left upper panel) or the whole lysates (input, right upper panel) with the V5 antibody. HER2 was detected in IPs (left lower panel) and input (right lower panel) using the 32H2 antibody that detects all forms of HER2. **b** IP against RANK-V5 was performed in HEK293 cells transfected with RANK-V5 and GFP, HER2-FLAG, a carboxy-terminal fragment of HER2 (611-CTF) or an amino-terminal fragment of HER2 (742-NTF) using the V5 antibody. In the IP and input, HER2 was detected using the 32H2 antibody. **c** IP against endogenous HER2 was performed in SKBR3 cells using trastuzumab (Herceptin-HCP) or a control IgG. Endogenous RANK and HER2 were detected in IP (RANK immunoprecipitated by HER2 is indicated by an asterisk (*) in the upper panel) and input samples

Due to the change in HER2 phosphorylation upon activation of RANK signaling with RANKL, we hypothesized that the two receptors might physically interact. To enable efficient immunoprecipitation and detection, we transiently co-expressed affinity-tagged versions of the receptors in HEK293 cells, including an amino (742-NTF) [30] and a carboxy-terminal fragment of HER2 (611-CTF) [31]. As shown in Fig. 6a, RANK-V5 was detected after immunoprecipitation of HER2 or 611-CTF HER2, but not in 742-NTF or any of the control samples (IgG), indicating that RANK interacts with the carboxy-terminal region of HER2. The reverse immunoprecipitation of RANK-V5 corroborated these results (Fig. 6b). To confirm the interaction between the two receptors under endogenous expression levels in the context of breast cancer, we chose SKBR3 cells that, compared to other breast cancer cell lines, express higher levels of HER2 and intermediate/lower levels of RANK and do not express EGFR [43]. HER2 was immunoprecipitated with the antibody trastuzumab (HCP) that binds to the HER2 extracellular domain, and the presence of RANK in the immunoprecipitate was tested by Western blotting. As seen in Fig. 6c, trastuzumab precipitated endogenous RANK demonstrating that the two receptors physically interact in breast cancer cells in an EGFR-independent manner.

## Discussion

A crosstalk between RANK and EGFR signaling has been described in the context of osteoclast differentiation [44], as well as in breast cancer for a particular RANK truncated isoform [45]. In the mammary gland, we found that pharmacological inhibition of RANKL decreases tumorigenesis and lung metastases in the MMTV-ErbB (Neu) transgenic mouse model [9]. In the same line, MMTV-ErbB mice with a heterozygous RANK deletion showed decreased pulmonary metastasis than RANK WT MMTV-ErbB controls [15]. In addition, RANKL treatment increased lung metastases in both FVB/N and MMTV-ErbB animals [15]. More recently, a review [46] followed by an article with experimental data [47], suggested the combination of RANK and HER2 signaling inhibition as a new strategy for the treatment of HER2-positive breast carcinomas.

In this study, we have shown that *RANK* gene expression increased after dual treatment with lapatinib and trastuzumab in HER2-positive tumor samples from the PAMELA clinical trial [22] and in HER2-positive breast cancer cell lines. These observations would point to increased RANK signaling in patients treated with anti-HER2 drugs. We also observed that the percentage of patients with RANK tumor expression doubled in the context of HER2 resistance when compared to treatment-naive HER2-positive breast tumors. Furthermore, both SKBR3- and BT474 HER2-positive cell lines with acquired lapatinib resistance displayed increased RANK expression and pathway activation compared to their respective lapatinib-sensitive controls. Thus, our combined analyses of HER2-positive breast cancer samples and cell lines demonstrate that RANK expression is higher in HER2-resistant breast cancer. RANK loss moderately sensitized lapatinib-resistant cells to the drug, and overactivation of RANK signaling increased lapatinib resistance in HER2-positive cell lines (SKBR3, BT474, and HCC1954). Based on these results, one could speculate that activation of RANK signaling may allow breast cancer cells to survive anti-HER2 therapies and the benefit of combining denosumab with HER2 inhibitors as postulated by [47].

NF-κB signaling has been shown to enhance ErbB2-induced tumor growth both in vitro and in immune-competent mice [48, 49]. Increased NF-κB activation downstream of RANK [50] may also contribute to lapatinib resistance. Hyperactive NF-κB signaling has been proposed as a possible resistance mechanism after lapatinib treatment in HER2-positive [51] and triple-negative breast cancer [52, 53]. In HER2-positive breast cancer, lapatinib-resistant cells show increased NF-κB levels and do not respond to single HER2 or NF-κB inhibitors, but to a combination of both [51]. The NF-κB expression is normally linked to invasive high-grade tumors, and several NF-κB inhibitors are currently being investigated [54, 55]. Chen and colleagues showed that lapatinib treatment induced a constitutive activation of NF-κB through Src-dependent p65 and IκBα phosphorylation, sensitizing the cells to proteasome inhibitors [52]; our data suggest that increased RANK being a well-known regulator of NF-κB may also play a role, although we cannot discard the contribution of other RANK-driven downstream pathways. The phosphorylation of IκBα, leading to its degradation and resulting in p50/p65 heterodimer nuclear translocation, is mediated by the IKK complex (comprising IKKα, IKKβ, and IKKγ/NEMO) [56, 57]. HER2 itself was shown to activate NF-κB via the canonical pathway involving IKKα in HER2-positive and ER-negative breast cancer cells [58]. IKKα also mediates NF-κB activation in mammary cells during pregnancy and after RANKL stimulation [59]. In our study, we did not observe clear changes in p65 phosphorylation after stimulation with ErbB ligands and the treatment with lapatinib could not counteract p65 phosphorylation driven by RANKL treatment in RANK-overexpressing HER2-positive cell lines, providing an alternative survival path for these cells.

Importantly, we have shown RANK binding to HER2 by co-immunoprecipitation experiments. Accordingly, Zoi et al. recently showed the interaction of RANK with ErbB family members by proximity ligation assays [47]. In this publication, the authors claim that the number of RANK/HER2 dimers in cells correlates with HER2 expression levels. Also, denosumab, trastuzumab, and/or pertuzumab treatment reduces the number of RANK/HER2 dimers whereas RANKL stimulation leads to an increased number of RANK/HER2 dimers [47]. Finally, their data show that RANKL addition decreases the efficacy of HER2 inhibitors [47]. In our hands, a direct interaction between RANK and HER2, independent of EGF, was observed. RANKL stimulation of HER2-positive breast cancer cells overexpressing RANK decreases HER2 phosphorylation, indicating that RANKL influences ErbB2 signaling.

RANKL was shown to promote migration in breast cancer cells after activation of the ERK and AKT pathways [60]. We have also found increased phosphorylation of ERK1/2 and AKT after RANKL treatment in SKBR3 and BT474 cell lines, with either physiological or increased RANK levels by receptor overexpression. Interestingly, we observed that RANKL-mediated induction of ERK1/2 and AKT phosphorylation was completely abrogated after lapatinib treatment in SKBR3 and BT474 cells, again independently of RANK receptor expression levels. These observations and the fact that RANK and HER2 interact suggest that lapatinib inhibits not only EGFR/HER2 tyrosine phosphorylation but also RANK signaling driven by RANKL (e.g., ERK1/2 and AKT). Importantly, in addition to the direct interaction between RANK and HER2, we observed that RANK signaling is functionally linked to the ErbB2 pathway. Additional research is required to address whether the direct RANK/HER2 interaction contributes to the enhanced resistance to lapatinib observed after activation of RANK signaling.

Taken together, we showed that anti-HER2 treatment and resistance acquisition both raised RANK expression levels in HER2-positive clinical breast tumors and cell lines. Also, enhanced RANK signaling increased lapatinib resistance in HER2 breast cancer cells. We found that RANK and HER2 physically and functionally interact. Altogether, these results hint to a dual RANK and HER2 inhibition therapy for RANK-expressing HER2-positive breast cancer patients, whose benefit remains to be tested.

## Conclusions

In summary, we showed that RANK is expressed in HER2-positive breast cancer samples, particularly in patients resistant to anti-HER2 blocking therapy. The RANK expression is often associated with younger age, hormone receptor-negative status, and higher histological grade and proliferation index. Moreover, in HER2-positive breast cancer samples from the PAMELA trial, RANK expression increased upon treatment with lapatinib and trastuzumab. This was confirmed in vitro in several HER2-positive human breast cancer cell lines suggesting that RANK signaling may contribute to the development of lapatinib resistance. Indeed, RANK-overexpressing HER2-positive cell lines showed increased resistance to lapatinib and higher NF-κB pathway activation. Finally, we demonstrated that RANK physically and functionally interacted with HER2 suggesting a RANK/HER2 crosstalk. Together, these results suggest that inhibition of RANK signaling may improve the response to anti-HER2 therapies in RANK-positive, HER2-positive breast cancer patients.

## Supplementary Information

**Additional file 1: Figure S1.** TMA H-scores and controls. A. RANK and RANKL H-scores in HER2-positive breast cancer samples, treatment-naïve (left panels) or anti-HER2-resistant (right panels). In treatment-naïve TMAs, each number represents a “core” from a single patient. In anti-HER2-resistant TMAs, scored independent tumor cores are numbered for each patient (after the symbol #). B. Representative pictures of human breast tumors from patient-derived xenografts used as positive and negative controls for RANK and RANKL IHC.

**Additional file 2: Figure S2.** RANK and RANKL staining in TMAs. A. Pictures of RANK and RANKL protein expression analyzed by IHC in the TMA cores from the treatment-naïve cohort. B. Pictures of RANK and RANKL protein expression analyzed by IHC in the TMA cores from the anti-HER2 resistant cohort.

**Additional file 3: Figure S3.** *RANK* and *RANKL* expression in breast cancer samples from the PAMELA clinical trial. A. Expression of *RANK* and *RANKL* across the intrinsic molecular subtypes from the PAMELA study. *P* values were calculated by comparing mean values across all groups. B. Scatter plots of *RANK* and *RANKL* expression versus *ERBB2* expression for baseline samples in the PAMELA study. Solid line in each figure represents the regression line. Pearson correlation coefficient (r) with significance (*p* value) is presented in each figure. C. Pearson correlation between single genes and gene expression signatures evaluated in baseline samples from the PAMELA study.

**Additional file 4: Figure S4.** *RANK* but not *RANKL* expression increased after dual anti-HER2 therapy in HR+ and HR- patient samples (*n* = 151) from the PAMELA trial. **A** and **B**. Ladder plots (left panels) showing *RANK* and *RANKL* gene expression in PAMELA HER2-positive HR+ (A) and HR- (B) tumors before (baseline) and after (surgery) dual anti-HER2 treatment. An increase in gene expression is represented in red and a decrease in blue. Each line represents a tumor sample from one patient. P values in A were calculated by comparing mean values between both groups and in B were determined by paired two-tailed t-tests. Density plots (right panels) showing *RANK* and *RANKL* gene expression in PAMELA HER2-positive HR+ (A) and HR- (B) tumors before (baseline) and after (surgery) treatment.

**Additional file 5: Figure S5.** **A**. Relative number of living (relative survival) SKBR3 and SKLR control cells incubated for 4 days with the indicated concentrations of lapatinib and stimulated with RANKL. Cells were seeded in growth media, 100 ng/ml RANKL were added 24h after seeding, lapatinib was added 24 h later and cells were analyzed with CCK8 as detailed in methods. Determinations were done in triplicates, mean values are depicted from *n* = 5 independent experiments and SD and *p*-value (**) calculated by one-way ANOVA is depicted (*p* ≤ 0.05 for SKBR3 vs SKLR and SKLR +RANKL, SKBR3 +RANKL vs SKLR and SKLR +RANKL; n.s. for SKBR3 vs SKBR3 +RANKL and SKLR vs SKLR +RANKL). Significance of relative survival was calculated for each concentration using two-tailed *p* values for one sample t test. RANKL significantly increased survival of SKLR cells at 0.018 μM of lapatinib (*p* = 0.019). **B.** Western blot showing the total levels of IκB**,** p65, ERK1/2, AKT and HER2 in SKBR3 control, SKLR control and SKLR sh#3 cells treated with RANKL or lapatinib as depicted in Fig. 4c. Cells were serum starved for 12 h and then treated with lapatinib (2 h) or RANKL (10 min) before processing them. Tubulin was used as a loading control. **C**. Table depicting the relative phospho-protein levels of the indicated proteins from the western blots shown in Fig. 4c and Fig. S5B determined by densitometry analyses with Image J.

**Additional file 6: Figure S6.** **A**. Relative number of living  (relative survival) SKBR3 RANK cells stimulated with RANKL in the presence or absence of denosumab (DNS) and incubated for 4 days with the indicated concentrations of lapatinib. Cells were seeded in growth media with/without denosumab (1 μg/ml), lapatinib was added after 24 h stimulation with 100 ng/ml RANKL, and cell viability was analyzed with CCK8 as detailed in methods. Determinations were done in triplicates, mean values from *n* ≥ 2 independent experiments and SD are depicted. **B.** Western blot analyses of total levels of p65, ERK1/2 and HER2 in whole lysates from SKBR3, BT474 and HCC1954 cells stably transduced with control (empty) or RANK overexpressing (RANK) vectors as depicted in Fig. 5d. Before collecting the cells, they were cultured in media without FBS for 12 h, pretreated with/without lapatinib for 2 h followed by 10 min stimulation with RANKL. Tubulin was used as a loading control. **C**. Table depicting the relative phospho-protein levels of the indicated proteins from the western blots shown in Fig. 5d and Fig. S6B determined by densitometry analyses with Image J.

**Additional file 7: Figure S7.** Western blot analyses of HER2 (p-HER2, p-ERK1/2) and NF-κB (p-p65) pathway activation in SKBR3, BT474 and HCC1954 cells stably transduced with empty or RANK overexpressing (RANK) vectors. Cells were cultured in media without FBS for 12 h, followed by stimulation with EGF (100 ng/ml) (upper panels) or heregulin (HRG 10 ng/ml) (lower panels) for the indicated times. Tubulin was used as a loading control.

**Additional file 8:** Supplementary Table 1.

## Data Availability

Data generated during this study are included in this published article (and its supplementary information files), and datasets generated and analyzed supporting the findings of this study are available from the corresponding authors upon reasonable request.

## Abbreviations

EGFR: Epidermal growth factor receptor
ER: Estrogen receptor
HER2: Epidermal growth factor receptor 2
IHC: Immunohistochemistry
IP: Immunoprecipitation
NF-κB: Nuclear factor kappa B
PR: Progesterone receptor
RANK: Receptor activator of NF-κB
RANKL: Receptor activator of NF-κB ligand
RT-qPCR: Reverse transcriptase quantitative PCR
TMA: Tissue microarray
