# Supplementary material for: RANK signaling increases after anti-HER2 therapy contributing to the emergence of resistance in HER2-positive breast cancer
Source: Breast Cancer Res. 2021 Mar 30;23:42. doi: 10.1186/s13058-021-01390-2 (PMC8008631; doi:10.1186/s13058-021-01390-2)
Supplement: Supplementary file 1 [file 13058_2021_1390_MOESM1_ESM.docx]

Figure S1

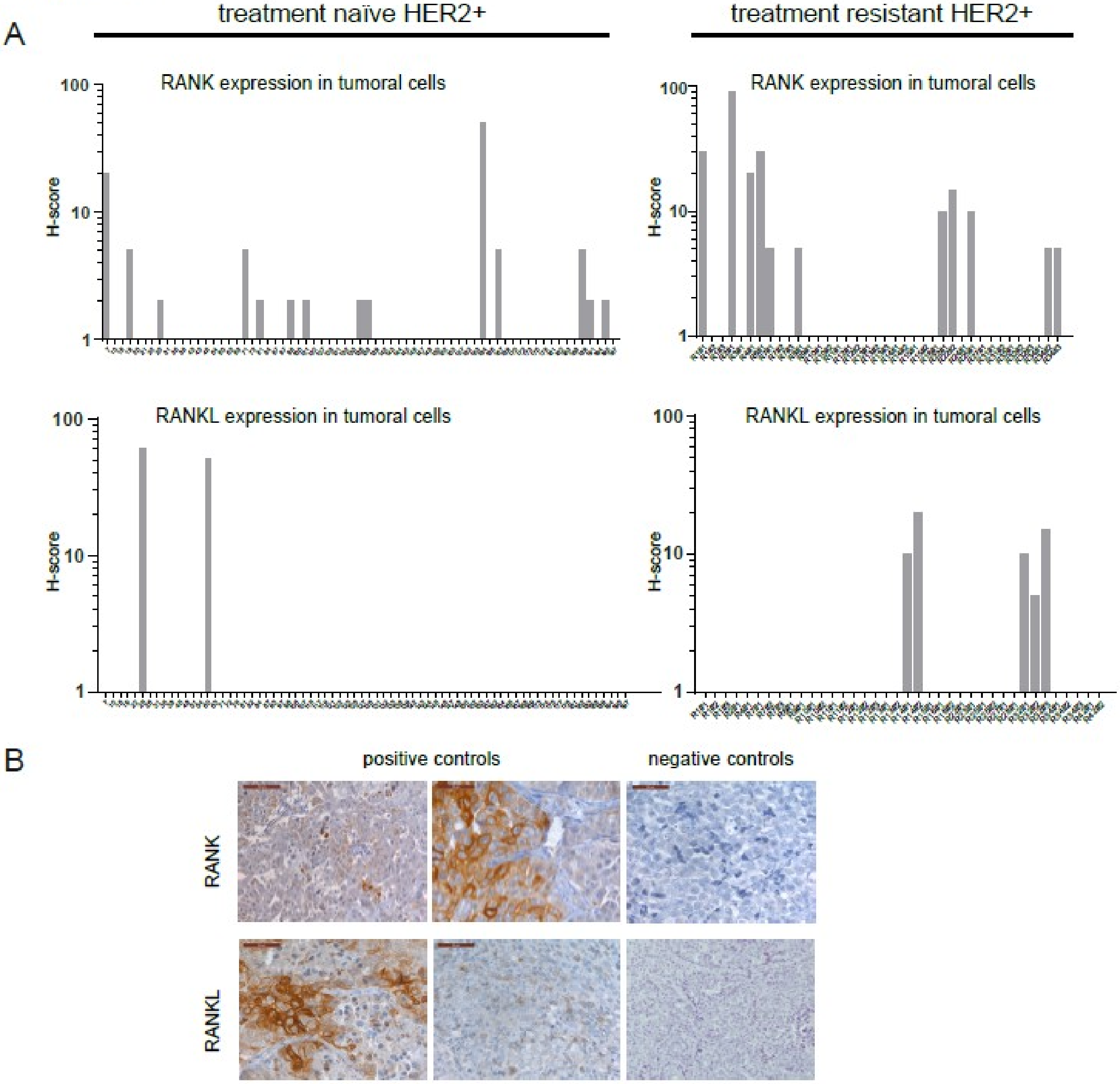
A
treatment naïve HER2+
treatment resistant HER2+
RANK expression in tumoral cells
RANKL expression in tumoral cells
H-score
100
10
1
B
positive controls
negative controls
RANK
RANKL
