# Supplementary material for: RANK signaling increases after anti-HER2 therapy contributing to the emergence of resistance in HER2-positive breast cancer
Source: Breast Cancer Res. 2021 Mar 30;23:42. doi: 10.1186/s13058-021-01390-2 (PMC8008631; doi:10.1186/s13058-021-01390-2)
Supplement: Supplementary file 3 [file 13058_2021_1390_MOESM3_ESM.docx]

Figure S3

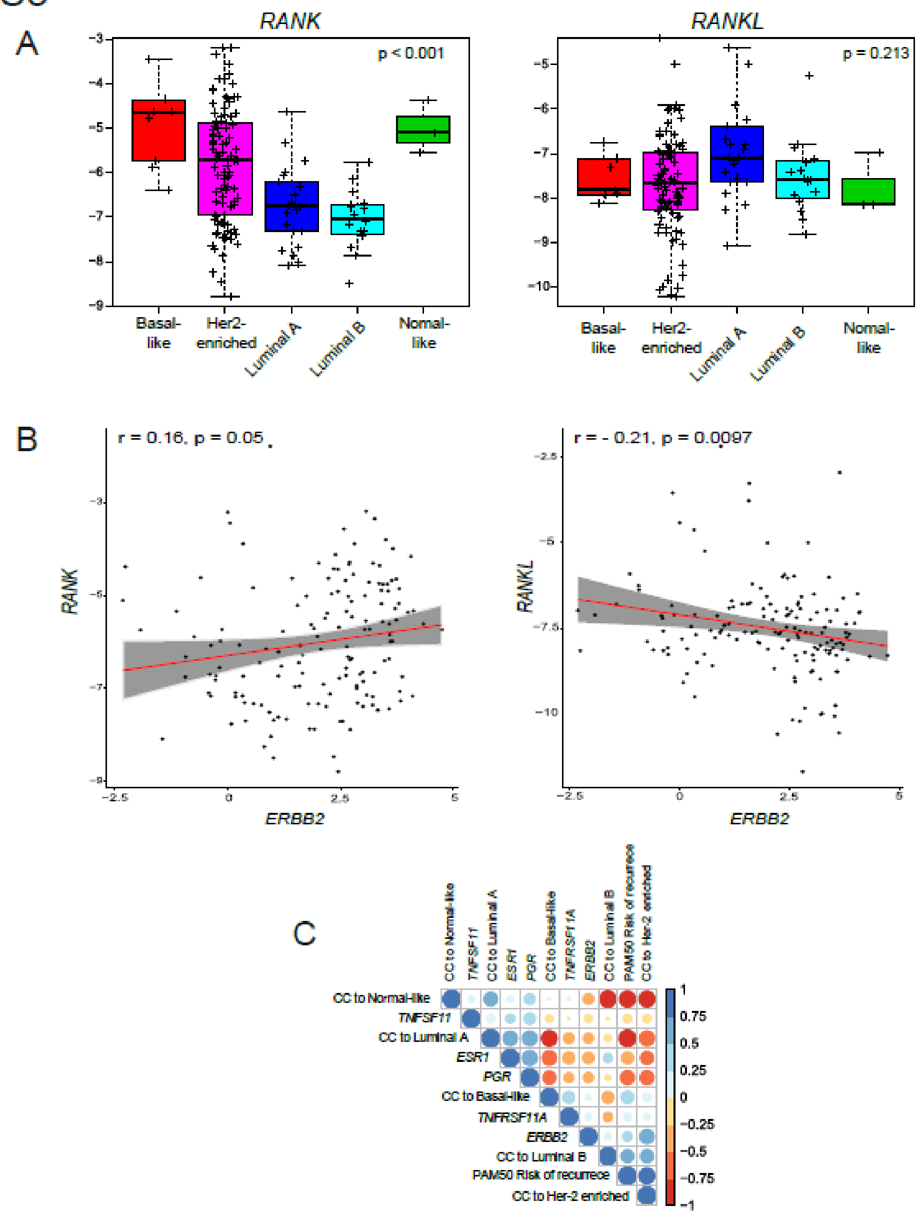
A
RANK
p < 0.001
Basal-like
Her2-enriched
Luminal A
Luminal B
Nomal-like
RANKL
p = 0.213
B
r = 0.16, p = 0.05
RANK
ERBB2
r = - 0.21, p = 0.0097
RANKL
ERBB2
C
CC to Normal-like
TNFSF11
CC to Luminal A
ESR1
PGR
CC to Basal-like
TNFRSF11A
ERBB2
CC to Luminal B
PAM50 Risk of recurrece
CC to Her-2 enriched
