# Supplementary material for: RANK signaling increases after anti-HER2 therapy contributing to the emergence of resistance in HER2-positive breast cancer
Source: Breast Cancer Res. 2021 Mar 30;23:42. doi: 10.1186/s13058-021-01390-2 (PMC8008631; doi:10.1186/s13058-021-01390-2)
Supplement: Supplementary file 4 [file 13058_2021_1390_MOESM4_ESM.docx]

Figure S4

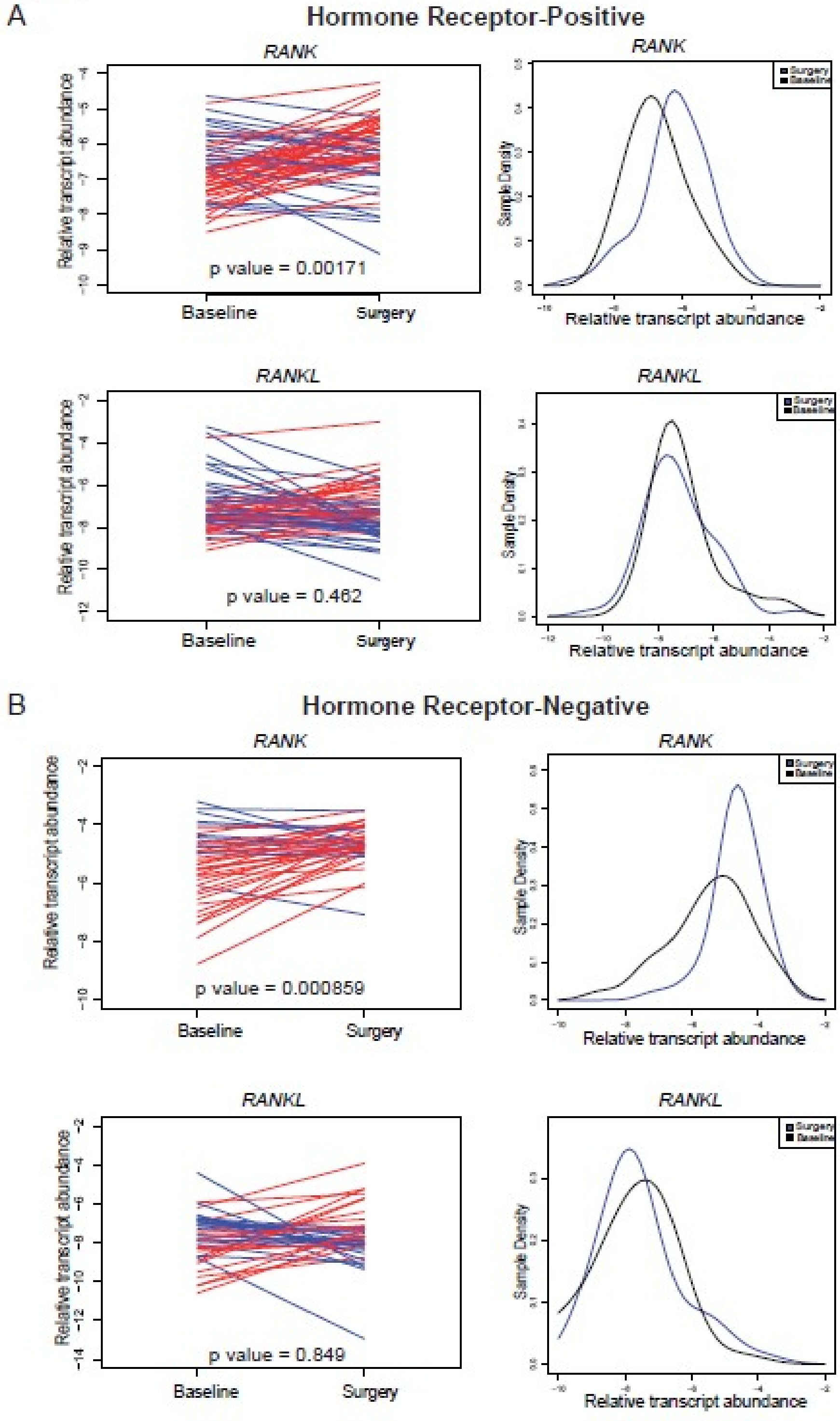
A
Hormone Receptor-Positive
RANK
Relative transcript abundance
p value = 0.00171
Baseline
Surgery
RANK
Surgery
Baseline
Sample Density
Relative transcript abundance
RANKL
Relative transcript abundance
p value = 0.462
Baseline
Surgery
RANKL
Surgery
Baseline
Sample Density
Relative transcript abundance
B
Hormone Receptor-Negative
RANK
Relative transcript abundance
p value = 0.000859
Baseline
Surgery
RANK
Surgery
Baseline
Sample Density
Relative transcript abundance
RANKL
Relative transcript abundance
p value = 0.849
Baseline
Surgery
RANKL
Surgery
Baseline
Sample Density
Relative transcript abundance
