# Supplementary material for: RANK signaling increases after anti-HER2 therapy contributing to the emergence of resistance in HER2-positive breast cancer
Source: Breast Cancer Res. 2021 Mar 30;23:42. doi: 10.1186/s13058-021-01390-2 (PMC8008631; doi:10.1186/s13058-021-01390-2)
Supplement: Supplementary file 5 [file 13058_2021_1390_MOESM5_ESM.docx]

Figure S5

A

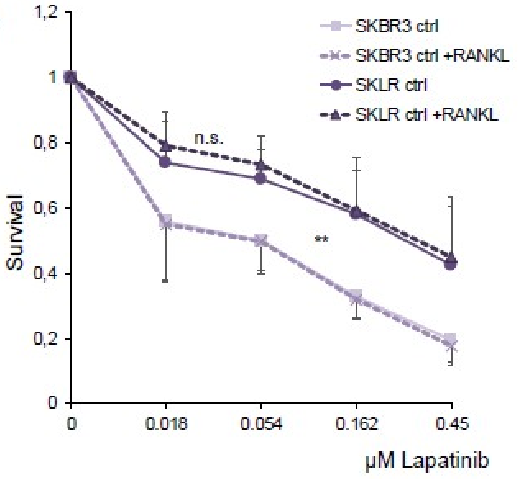

B

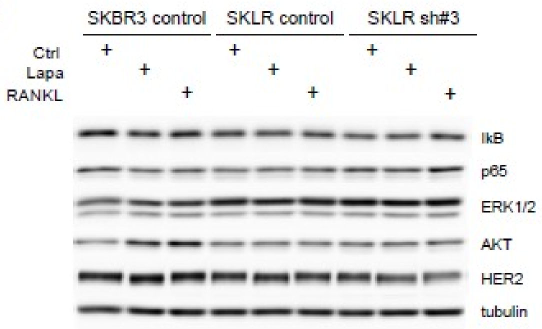

C

	SKBR3 control			SKLR control			SKLRsh#3		
	control	Lapa	RANKL	control	Lapa	RANKL	control	Lapa	RANKL
p-IkB	1,0	0,5	2,0	1,6	1,5	3,5	2,7	1,5	2,2
p-p65	1,0	0,7	1,5	1,5	1,4	2,4	1,8	0,8	1,7
p-ERK1/2	1,0	0,0	0,6	1,0	0,0	1,0	0,3	0,0	0,6
p-AKT	1,0	0,0	0,5	0,7	0,0	0,9	0,6	0,0	1,0
p-HER2	1,0	0,0	1,0	0,6	0,0	0,7	0,6	0,0	0,7
