# Supplementary material for: RANK signaling increases after anti-HER2 therapy contributing to the emergence of resistance in HER2-positive breast cancer
Source: Breast Cancer Res. 2021 Mar 30;23:42. doi: 10.1186/s13058-021-01390-2 (PMC8008631; doi:10.1186/s13058-021-01390-2)
Supplement: Supplementary file 6 [file 13058_2021_1390_MOESM6_ESM.docx]

Figure S6

A

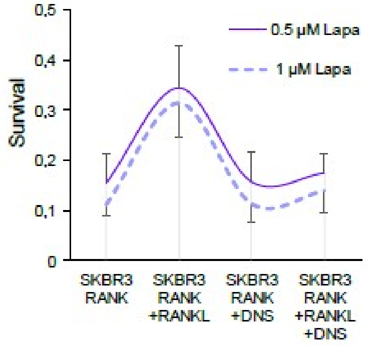

B

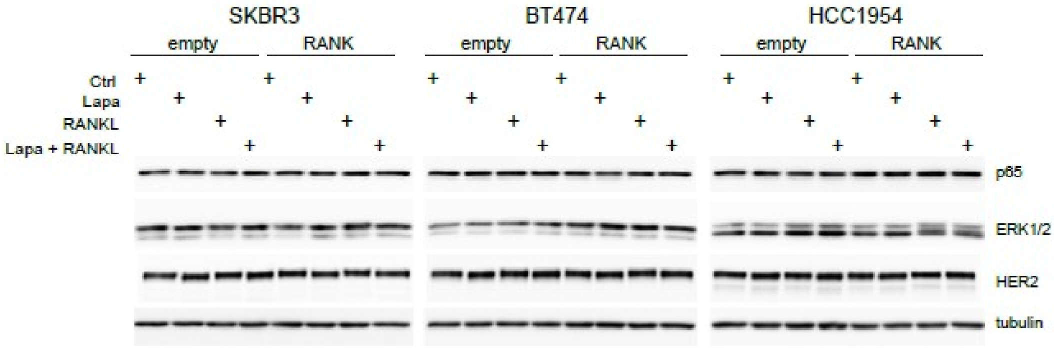

C

	SKBR3 empty				SKBR3 RANK				BT474 empty			
	control	Lapa	RANKL	Lapa + RANKL	control	Lapa	RANKL	Lapa + RANKL	control	Lapa	RANKL	Lapa + RANKL
p-p65	1,0	0,9	1,7	1,8	3,1	3,0	31,3	31,3	1,0	1,1	0,8	0,5
p-ERK1/2	1,0	0,0	1,0	0,0	2,0	0,0	4,1	0,0	1,0	0,1	1,2	0,1
p-AKT	1,0	0,0	1,8	0,0	1,4	0,0	2,1	0,0	1,0	0,1	1,2	0,1
p-HER2	1,0	0,0	1,2	0,0	1,3	0,0	1,1	0,0	1,0	0,0	0,9	0,0

	BT474 RANK				HCC1954 empty				HCC1954 RANK			
	control	Lapa	RANKL	Lapa + RANKL	control	Lapa	RANKL	Lapa + RANKL	control	Lapa	RANKL	Lapa + RANKL
p-p65	0,5	1,5	69,7	43,2	1,0	1,0	6,1	7,4	2,7	2,0	29,5	27,5
p-ERK1/2	0,5	0,1	1,6	0,1								
p-AKT	0,5	0,1	1,6	0,1	1,0	0,1	2,8	0,1	2,3	0,2	3,0	0,5
p-HER2	1,6	0,0	0,8	0,0	1,0	0,0	0,8	0,0	0,5	0,0	0,2	0,0
