# Supplementary material for: RANK signaling increases after anti-HER2 therapy contributing to the emergence of resistance in HER2-positive breast cancer
Source: Breast Cancer Res. 2021 Mar 30;23:42. doi: 10.1186/s13058-021-01390-2 (PMC8008631; doi:10.1186/s13058-021-01390-2)
Supplement: Supplementary file 7 [file 13058_2021_1390_MOESM7_ESM.docx]

# Figure S7

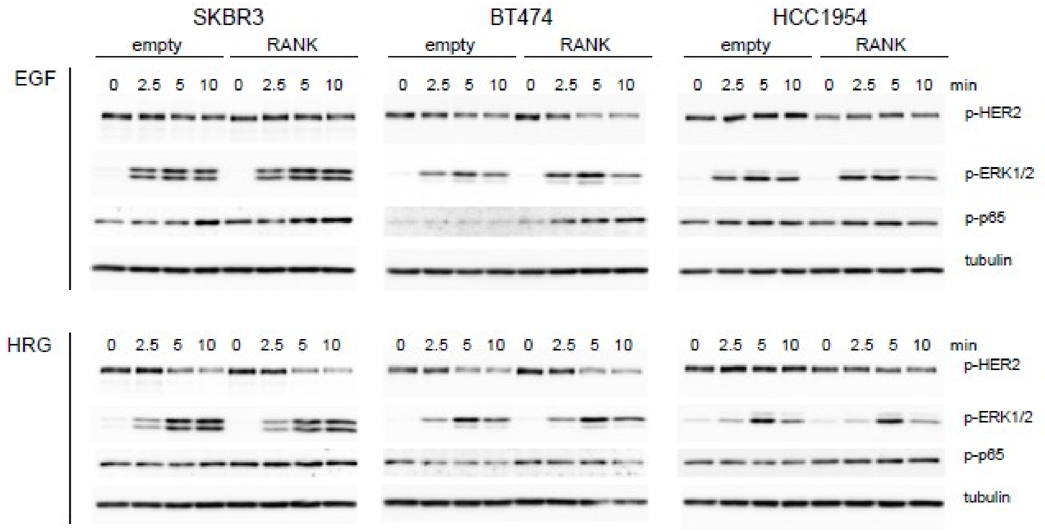
SKBR3
BT474
HCC1954
empty
RANK
EGF
HRG
0 2.5 5 10 0 2.5 5 10
min
p-HER2
p-ERK1/2
p-p65
tubulin
